# Refractory post-surgical cystoid macular edema managed following suprachoroidal microcatheterization and delivery of triamcinolone

**DOI:** 10.1186/s12886-023-03110-0

**Published:** 2023-09-05

**Authors:** Marc D. de Smet, Matthieu Goncerut, Friedrich Asmus, Ron Yamamoto

**Affiliations:** 1 Helvetia Retina Associates, Lausanne, Switzerland; 2 New York Eye and Ear Infirmary of Mt Sinai, Icahn School of Medicine, New York City, NY USA; 3 Oxular Ltd, Oxford, UK

**Keywords:** Microcatheterization, CME, Drug visualization, Infrared, Macular edema, OCT, Posterior, Steroid, Suprachoroidal

## Abstract

**Background:**

Post-surgical macular edema (ME) is a common cause of prolonged visual impairment. Here we report on the feasibility and clinical outcomes from the use of a novel suprachoroidal microcatheter to treat post-surgical chronic ME by the posterior suprachoroidal placement of a triamcinolone acetonide (TA) suspension.

**Methods:**

Two patients were catheterized with the Oxulumis suprachoroidal delivery system on two separate occasions starting 5 and 10 mm posterior to the limbus. The catheter only remains in the suprachoroidal space for the time of the drug administration. Visual acuity and spectral domain optical coherence tomography (SD-OCT) changes were followed over several weeks to months to determine the duration of ME resolution.

**Results:**

Suprachoroidal microcatheterization for posterior delivery of triamcinolone was possible in all attempts using the illuminated Oxulumis catheter. No reflux, scleral or choroidal trauma was observed. There was no intraocular pressure rise during the follow-up period. The triamcinolone deposit was visible on infrared imaging and on SD-OCT a choroidal elevation was visible. Both progressively disappeared over time. A rapid resolution of ME associated with improved vision was observed following each injection for 3 to 7 months with a TA dose of 2.4 mg or 4 mg.

**Conclusions:**

In these patients with poorly responsive ME, posterior suprachoroidal TA led to a visible suprachoroidal drug deposit and prolonged visual improvement. The Oxulumis microcatheterization device performed as expected and was not associated with any complications.

## Background

Autoimmune or post-surgical macular edema (ME) are common causes of vision loss. In addition, 60% of patients with birdshot retinochoroiditis, sarcoidosis or intermediate uveitis with a duration of more than 1 year have ME [1]. Prevalence of ME increases with duration of inflammation, and chronic ME is associated with permanent loss of vision [2, 3]. Following surgery, ME is seen in 15% of patients having undergone a macular peel, [4] and ME after cataract surgery is observed in up to 11% of patients irrespective of underlying pathology [5]. Underlying mechanisms of ME include the release of prostaglandins from iris manipulation, an inflammation-driven alteration of aquaporin channels in Mueller cells, and loss of tight junctions in the retinal pigment epithelium (RPE), all leading to an altered water balance in the fovea [1]. Several strategies have been devised and tested to restore the balance of water flow in and out of the retina and thus eliminate edema both anatomically and angiographically, with varying degrees of success.

Some therapeutic approaches such as acetazolamide and somatostatin are aimed at improving the RPE pump [6, 7]. Others target one or more of the inflammatory mediators [8]. Steroids remain the most effective modality as they act on multiple inflammatory pathways while also inhibiting vascular leakage. Their effectiveness is determined by their local concentration. Sufficient levels of steroids are required in the macular area for them to be effective [9]. This is also their limitation, as both systemic and local steroids are associated with a multitude of side effects [10]. Steroids are known to induce cataracts and glaucoma with a risk that varies based on the agent used, exposure, the mode of delivery, and proximity to the target tissue [11, 12]. Subtenon delivery can result in sufficient steroid levels in the choroid and retina, with lower levels appearing in the vitreous and anterior structures compared with intravitreal (IVT) delivery [9, 13]. The reported rate of raised intraocular pressure in the absence of reflux and of cataract is also lower than following IVT injections, despite macular steroid doses that are 10 times higher compared with IVT delivery [14, 15]. Technical challenges and outcomes with intravitreal injections are well described and summarized in the review article by Meyer et al. [16].

An innovative alternative approach which achieves high levels of therapeutic agent in the retina and choroid consists of placing the steroid into the suprachoroidal space [17]. As the suprachoroidal space is virtual, specifically designed medical devices are needed to allow routine drug administration. One recent approach uses a microneedle of 0.9 or 1.1 mm length with anterior insertion at the pars plana and a “loss-of-resistance” method to inject in the suprachoroidal space. A number of recent clinical trials with a soluble form of triamcinolone acetonide (TA) have been conducted [18–23]. In the DOGWOOD trial, injection of 4 mg of triamcinolone into the peripheral suprachoroidal space using this microneedle led to a significant reduction in retinal thickness in 56% (9 of 16) of eyes at 2 months in patients with uveitis (88% posterior or intermediate) [18]. An alternative may be the use of a triamcinolone suspension which, following a suprachoroidal injection, tends to remain close to the site of injection, with limited diffusion [24]. This may require a more posterior placement to provide a greater and more sustained effect. To this aim, the current report describes clinical experience in the context of compassionate use in the first two patients treated with a novel ophthalmic administration device targeting the posterior suprachoroidal space with a microcatheter. This device uses a flexible illuminated microcatheter semi-automatically inserted into the suprachoroidal space to place triamcinolone at or beyond the equator in patients with inflammatory or post-surgical ME unresponsive to standard therapy. The microcatheter is withdrawn after completion of the drug injection. A more posterior placement was associated with the visualization of the triamcinolone on OCT and infrared imaging, allowing us to monitor the progressive disappearance of the product and the duration of effect.

## Methods

Two patients with post-surgical inflammatory ME unresponsive to standard intraocular or periocular therapy were treated in the context of compassionate use following a physician’s request. Oxular Ltd (Oxford, UK) agreed on a per patient, per treatment basis to provide the ophthalmic administration device. For compassionate use, a formal ethics approval was deemed unnecessary by the Comité d’Éthique Médicale du Canton de Vaud, as Triesence is approved for use in Switzerland. Approval for the use of the Oxulumis device was obtained from SwissMedic under the provision that it was used with an approved medical product. All patients provided written informed consent.

**Fig. 1:**
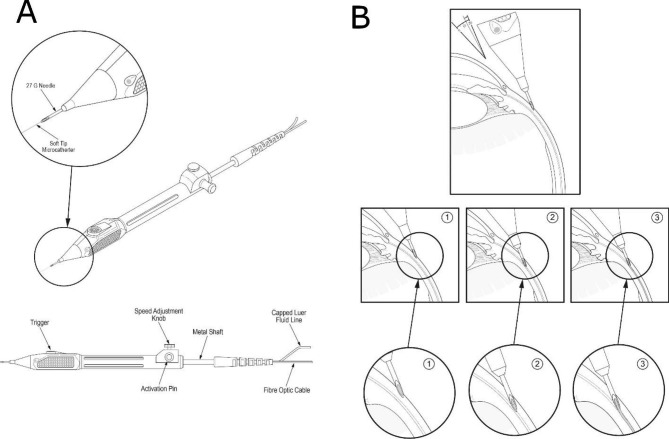
(**A**) Schematic of the Oxulumis device for suprachoroidal catheterization and drug delivery. Depression of the lever allows deployment of an illuminated flexible catheter. (**B**) Schematic of the site of insertion and deployment of the Oxulumis device. The insertion site is at or beyond the pars plana, posterior to the limbus (left). The needle is inserted initially with an angle between 40 and 45 degrees until the bevel is fully engaged. The angle is then reduced to 15–20 degrees relative to the surface of the sclera (1). Even with the microcatheter triggered (2), deployment into the suprachoroidal space only happens once the needle tip has advanced past the scleral wall (3)

**Fig. 2:**
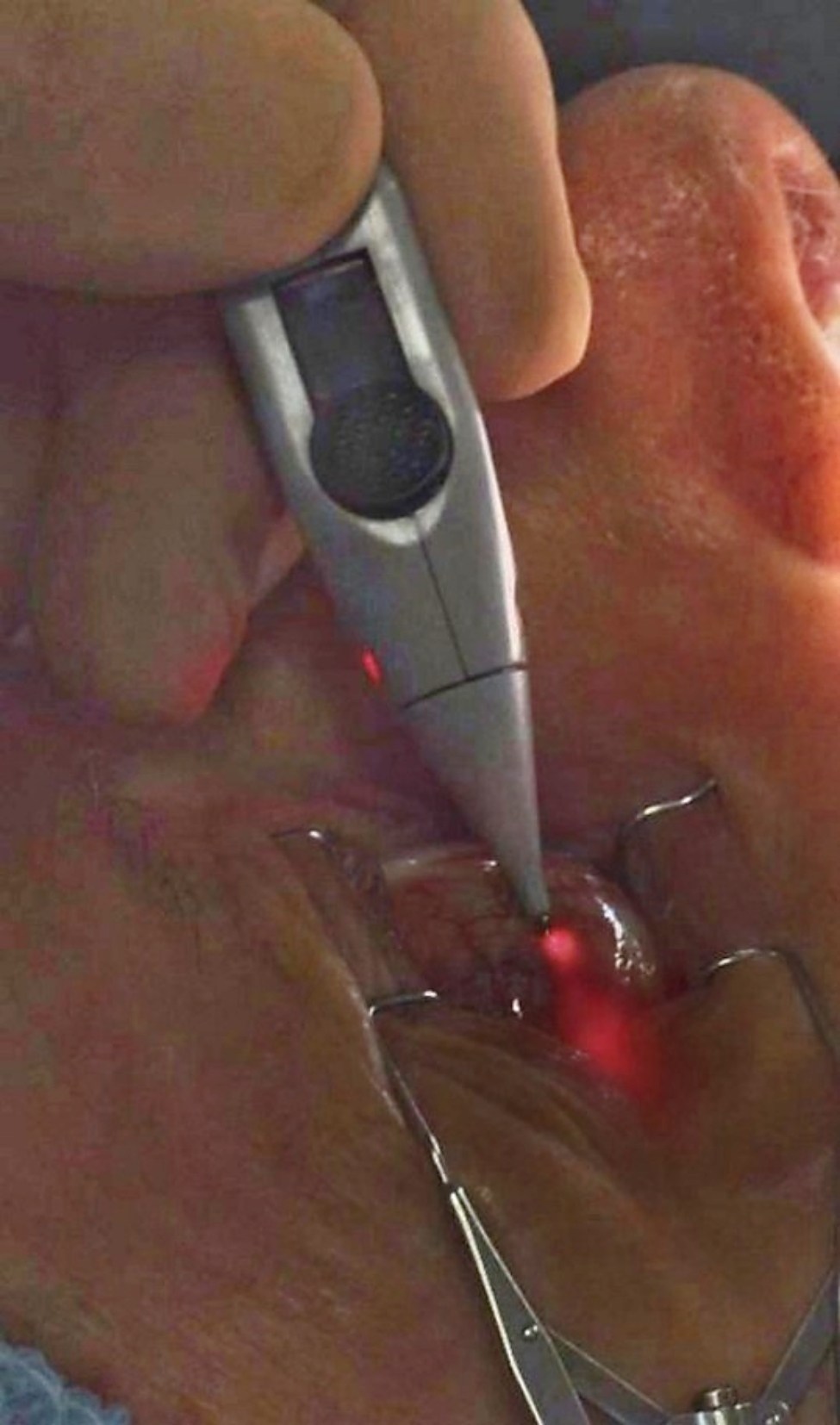
Image of the clinical deployment of the Oxulumis device in the suprachoroidal space. The red light from the illuminated microcatheter is visible through the sclera and confirms correct suprachoroidal placement

Patients were injected with an aqueous suspension of TA (40 mg/mL; Triesence, Novartis) through an Oxulumis Ophthalmic Administration Device (Oxular Ltd, Oxford, UK). The Oxulumis Ophthalmic Administration Device is a semi-automated device designed to deploy an illuminated ophthalmic microcatheter into the suprachoroidal space (Fig. 1A). The device consists of a 27-gauge insertion needle containing a flexible microcatheter. The needle is inserted with an initial angle of about 45 degrees until the bevel is fully engaged. Following the activation of the trigger, the needle is advanced tangential to the sclera, parallel to one of the rectus muscles. After triggering the release of the catheter, it remains under tension, held back by the sclera which acts as a ‘biological seal’ (Fig. 1B). Advancing the device further allows the tip of the insertion needle to pass through the scleral wall. When the needle tip just reaches the suprachoroidal space, the Oxulumis microcatheter is semi-automatically deployed to the suprachoroidal space driven by a spring inside the device. The Oxulumis microcatheter is composed of a flexible polymer with a distal tip configured for atraumatic deployment into the suprachoroidal space. The illuminated microcatheter is visible through the sclera, thus providing confirmation that the microcatheter is deployed correctly prior to the administration of triamcinolone (Fig. 2). If the illuminated microcatheter is not visible (placement is too deep, i.e. vitreal deployment) or directly visible (too superficial, i.e. subconjunctival deployment), the procedure can be interrupted and re-attempted in the same or another location. The steroid administration is only performed after confirming that the microcatheter is successfully deployed in the suprachoroidal space.

Both patients were injected twice. At the first microcatheterization, the catheter insertion point was 5 mm posterior to the limbus and 60 µl/2.4 mg of TA was injected. When the second treatment was performed, the insertion point was 10 mm posterior to the limbus and 100 µl/ 4 mg of TA was administered. Prior to the catheterization, a subtenon injection of lidocaine 2% was given away from the site of suprachoroidal injection.

Following appropriate deployment, the administration was performed by continuous, slow injection over a 30-second period, followed by a 30-second rest period before retracting the catheter. The sclera was examined for any sign of damage or reflux. Using the indirect ophthalmoscope, the retina was examined for any sign of damage, and for the presence of a choroidal elevation in the area of the injection. The intraocular pressure was measured 15 min after completing the treatment. Before treatment and during the post-treatment follow-ups, the following information was documented: best corrected visual acuity, intraocular pressure, macular volume SD-OCT scan (Spectralis, Heidelberg Engineering, Heidelberg, Germany), and an Optos colour image of the area of injection. If the site of injection was visible with the Spectralis 55 degree lens, the site of the suprachoroidal injection was documented by SD-OCT and with infrared imaging as the triamcinolone deposit was visible on infrared images. Patients were seen for follow-ups to monitor the compassionate use in the practice: every 2 weeks for the first month, then monthly to observe and detect any sustained visual acuity drop, an increase in the macular volume on OCT, or the appearance of intraretinal cysts.

## Results

**Fig. 3:**
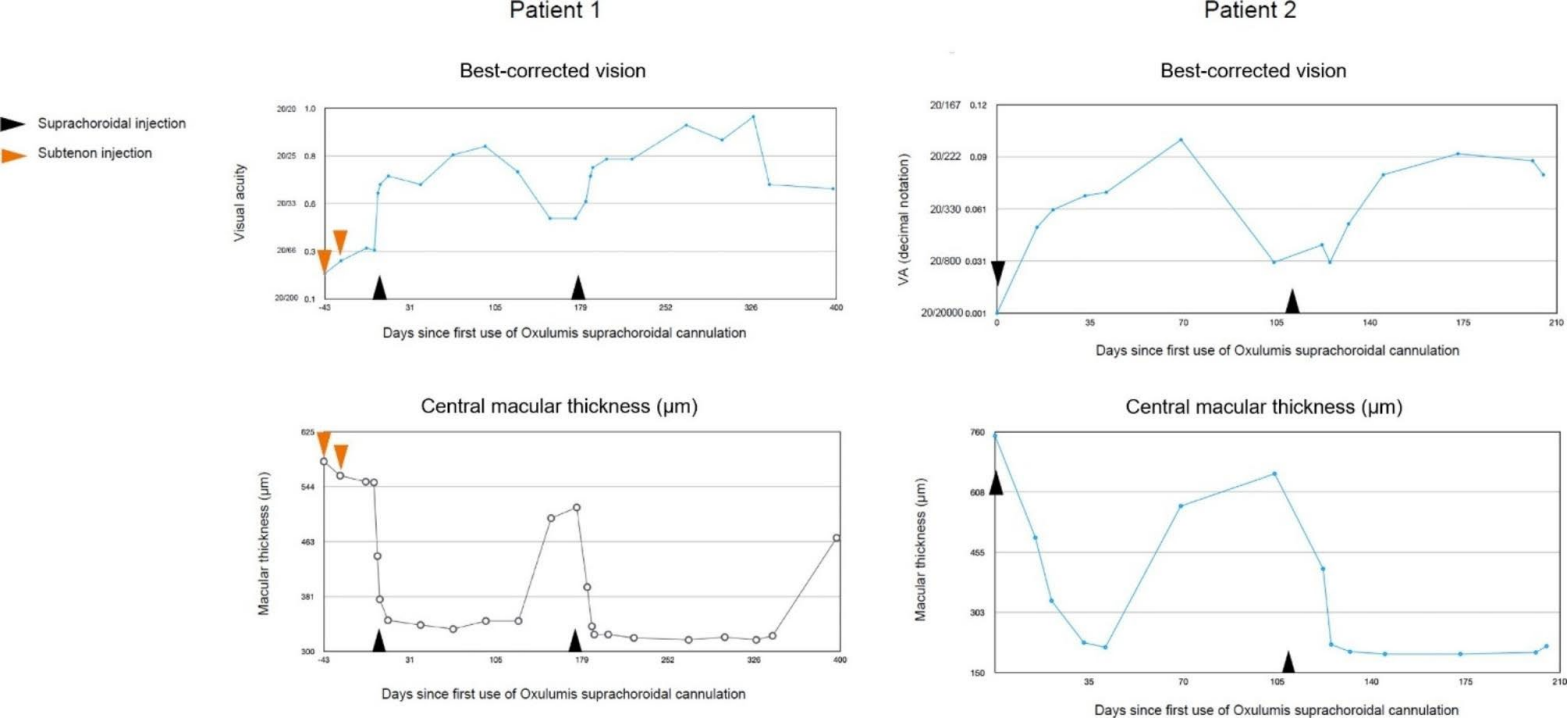
Evolution of visual acuity and OCT central macular thickness over time in Patient 1 and Patient 2. The time points of suprachoroidal injection are indicated by black triangles (days 0 and 183 for Patient 1, and days 0 and 107 for Patient 2). In Patient 1, two subtenon injections at days − 43 and − 29 are indicated by an orange arrow

The first patient was a woman above 75 years old who suffered a dropped nucleus during cataract surgery. A 3-piece intraocular lens (IOL) was repositioned in the sulcus and sutured. Vision was initially restored to 1.0 (20/20). A year later, vision was 0.2 (20/50), and anterior segment cells and visually significant ME were evident. Malposition of the haptics was suspected within the angle. The lens was replaced with a scleral fixation lens (FIL SSF; Soleko, Milan, Italy) positioned 3 mm posterior to the limbus. Despite topical steroid drops, a low grade anterior inflammation persisted between 0.5 and 1 + cells, while SD-OCT showed persistent ME with a central macular thickness (CMT) fluctuating between 550 and 650 μm during the first 2 post-operative months. Two subtenon injections of triamcinolone were given, at 2 months and 2.5 months after the last surgery. A limited reduction in CMT was observed on SD-OCT (Fig. 3).

**Fig. 4:**
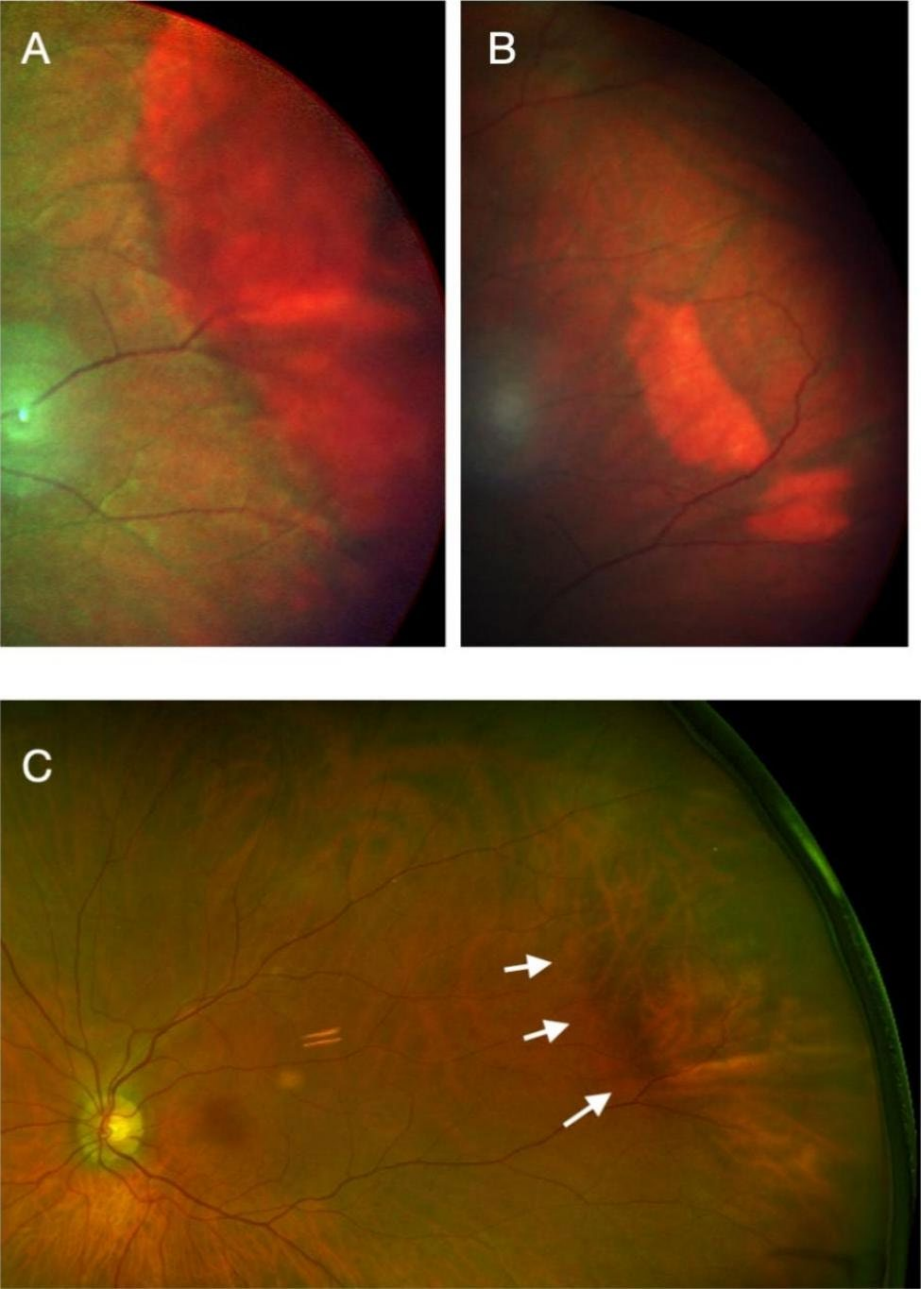
Heidelberg multispectral view of Patient 1: (**A**) at the time of the second injection, and (**B**) 118 days later. (**C**) shows the Optos image following the second injection. A temporal indentation is visible and delineated by the white arrows

**Fig. 5:**
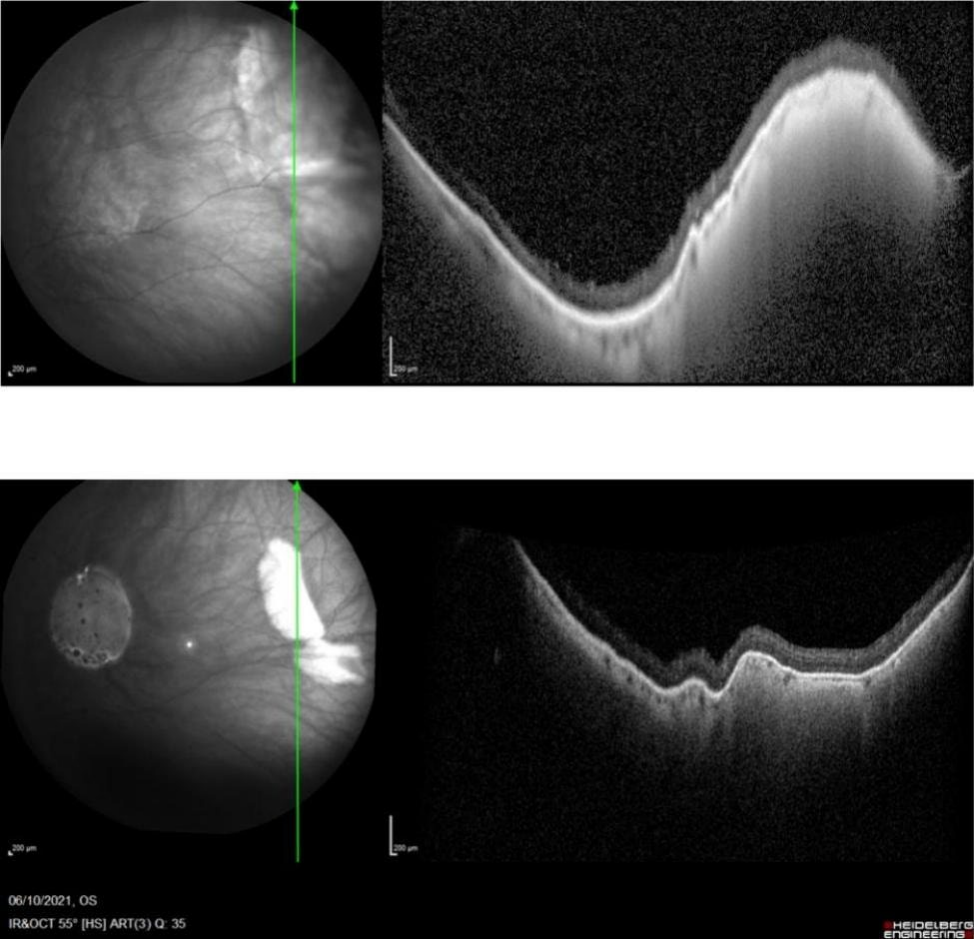
Heidelberg Spectralis imaging of the injection site in Patient 1: (**A**) at the time of the second injection and (**B**) 118 days later. The image on the left corresponds to the infrared imaging, while the image on the right is the corresponding enhanced depth imaging OCT image of the deposit. Both images show regression of the deposit over time

Four weeks after the last subtenon injection, 60 µL (2.4 mg) of TA was injected into the suprachoroidal space with the scleral insertion initiated 5 mm behind the limbus. Following this suprachoroidal microcatheterization, there was no subconjunctival hemorrhage, scleral damage or reflux. Upon indirect ophthalmoscopy, a choroidal elevation was noted slightly posterior to the equator of the eye in the meridian of the TA injection. There was no retinal damage. Intraocular pressure 15 min after the end of the microcatheterization procedure was normal. The retina was re-examined 3 days later, at which point the choroidal elevation had subsided. On SD-OCT, a choroidal elevation was noted in the area of the injection, as well as obscuration of the choroidal details underlying the area of elevation. It progressively disappeared over the following weeks. Infrared imaging taken of the area of elevation revealed the presence of TA visible as a white deposit in the choroid obscuring details of the underlying choroid. The obscuration and the material progressively disappeared over a period of 4 months. Following the microcatheterization, ME rapidly subsided with a few inner nuclear cysts persisting after 1 month. As typically observed in ME patients, vision improvement closely followed the changes in retinal thickness and ME (Fig. 3). Once the TA bleb in the suprachoroidal space had disappeared (after 4 months), ME increased and vision worsened. The patient was re-injected with 100 µl (4 mg) of TA, starting 10 mm posterior to the limbus. Initial anatomical and imaging observations were identical to the previous microcatheterization but with a more posterior location, about 5.5 disc diameters from the fovea (Fig. 4). The improvement in vision persisted for over 6 months (Fig. 3). The disappearance of the product on SD OCT was also clearly visible (Fig. 5).

The second patient, a man above 75 years old was on acenocoumarol (Sintrom; Medius AG, Muttez, Switzerland) following an aortic valve replacement and carotid end arterectomy. The eye of interest had suffered from a central retinal vein occlusion 1 year prior to a phacoemulsification procedure. During the procedure, a capsular rupture led to a fragment of the nucleus falling into the vitreous cavity. A floppy iris syndrome induced by an alpha-1 adrenergic inhibitor (Duodart; GlaxoSmithKline, Munchenbuchsee, Switzerland) caused an iris prolapse and pigment loss resulting subsequently in considerable glare. The IOL placed in the sulcus was partially dislocated. It was replaced by a scleral fixation lens (FIL SSF; Soleko, Milan, Italy), with anchors placed at 3 mm posterior to the limbus above a type 96 C Morcher ring (Morcher, Stuttgart, Germany) held in place above the remaining capsular support. Intraocular pressure increase due to iris neovascularization developed 2 months after the second surgery leading to additional panretinal laser with resolution of the neovascularization. A XEN implant (Allergan-Abbvie, Puurs, Switzerland) was inserted in the eye to control intraocular pressure. ME unresponsive to a 40 mg subtenon triamcinolone was noted shortly after the last surgical procedure. The first suprachoroidal injection consisted of 60 µl (2.4 mg) of TA, with the insertion point of the Oxulumis device for the first treatment at 5 mm posterior to the limbus, using an angle of approach parallel to the inferior rectus muscle. Similar post-suprachoroidal microcatheterization observations were recorded as in the first patient. The choroidal elevation initially seen after injection, located above the equator, was not present at the day 3 post-operative visit. Morphological and visual acuity improvements were observed, starting shortly after the microcatheterization and persisting for 3 months (Fig. 3). The second treatment using the Oxulumis device was performed using a scleral insertion starting 10 mm posterior to the limbus. Again, an improvement in vision was noted which persisted for another 3 months.

## Discussion

Routine suprachoroidal delivery of ophthalmic drugs has been an unmet need for retinal treatments since its first conceptualization in the 1960s. We report on two cases of safe and effective compassionate use of a novel semi-automated microcatheterization procedure to administer drugs to the posterior suprachoroidal space in a routine setting. Improved vision and reduction in ME were observed for up to 1 year in two patients with recalcitrant post-surgical ME. In the patients presented, ME did not respond to subtenon steroid injections, and intraocular/intravitreal sustained steroid implant injections were not an option due to the potential for anterior migration. Using 2.4 to 4 mg of TA, a prolonged therapeutic response with relevant vision gains lasted for 3 to 7 months. The Oxulumis procedure was safe with no relevant adverse events reported. Of note, one patient was on oral anticoagulation at the time of the Oxulumis procedure, a co-medication which would be generally expected to put patients at an increased risk for a suprachoroidal hemorrhage.

All procedures were carried out in a routine setting for a retinal specialist using a bed with a headrest and local subtenon lidocaine injections. The intervention time was short with total duration of a single microcatheterization lasting typically 3 to 5 min. No conclusion on the overall safety profile of the Oxulumis procedure is possible based on two compassionate use patients. This will require use by a larger group of retina specialists with differing levels of experience, and a larger number of patients.

With respect to the therapeutic effects observed, the second suprachoroidal microcatheterization in Patient 1, with a higher dose of 4 mg and a more posterior placement, led to a longer period during which edema was absent of 7 months. In the second patient, a more posterior placement did not lead to a prolonged resolution of the edema. While placing drugs closer to the posterior pole leads to higher foveal concentrations experimentally, further studies will be necessary to demonstrate if this is of clinical relevance with steroids.

In using the suprachoroidal microcatheterization procedure, the angle of approach for the initial insertion phase is tangential to the sclera. The semi-automated deployment of an illuminated spring-loaded catheter as the insertion needle passes through the sclera allows the retina specialist to concentrate on the correct insertion and depth of the needle. The illumination confirms the appropriate positioning without further manipulation prior to initiating the injection of triamcinolone. For aqueous suspension like TA, during the injection, the light flickers, providing further confirmation of an appropriate deployment and positioning. A momentary pause of 20–30 s prior to removing the catheter is helpful in preventing reflux after drug deployment. In the two cases reported here, catheterization of the suprachoroidal space was atraumatic, even in a patient on anticoagulation, with no adverse effect or bleeding noted. A more posterior catheterization at the equator of the eye was performed with equal ease. Neither patient experienced pain, though in both cases, a subtenon injection of anesthetic was given prior to the injection. Given that the DOGWOOD phase II and the PEACHTREE Phase III trials were carried out under topical anesthesia, it is possible that suprachoroidal catheterization could be done in a similar fashion in appropriate patients [18, 19].

On infrared imaging, triamcinolone was visible using infrared imaging as a white material obscuring the view of underlying choroidal structures. This was true whether the injection was initiated from a position 5 or 10 mm posterior to the limbus. Its progressive disappearance was associated with a decrease in the height of the choroidal deposit on a standard Heidelberg SD-OCT, and when the product had resorbed, the ME in both cases recurred. This may present an interesting approach to monitor response to treatment as it may allow treating physicians to predict when a patient may require further treatment based on the amount of residual drug present in the suprachoroidal space. No modification to existing OCT equipment is required, provided the product is injected sufficiently posteriorly in relation to the arcades.

## Conclusions

In conclusion, microcatheterization of the suprachoroidal space appears safe, and it was feasible to deliver a reflux-free deposit of triamcinolone in a posterior location, either equatorial or closer to the arcade based on the site of entry. The delivery is atraumatic, and the amount of residual drug can be monitored over time. Triamcinolone in this location has a profound effect on post-surgical ME resistant to more conventional means of therapy. These results are preliminary, performed by only one surgeon, and will need confirmation in a larger series of patients and retinal specialists in different treatment settings including in-clinic administration.

## Data Availability

The datasets generated and/or analysed during the current study are not publicly available in order to avoid compromising individual privacy but are available from the corresponding author on reasonable request.

## Abbreviations

CMT: Central macular thickness
IOL: Intraocular lens
IVT: Intravitreal
ME: Macular edema
OCT: Optical coherence tomography
RPE: Retinal pigment epithelium
SD-OCT: Spectral domain optical coherence tomography
TA: Triamcinolone acetonide
